# Lipidome alterations in human prefrontal cortex during development, aging, and cognitive disorders

**DOI:** 10.1038/s41380-018-0200-8

**Published:** 2018-08-08

**Authors:** Qianhui Yu, Zhisong He, Dmitry Zubkov, Shuyun Huang, Ilia Kurochkin, Xiaode Yang, Tobias Halene, Lothar Willmitzer, Patrick Giavalisco, Schahram Akbarian, Philipp Khaitovich

**Affiliations:** 1 Shanghai Institutes for Biological Sciences, Chinese Academy of Sciences, Shanghai, 200031 China; 2 CAS Key Laboratory of Compstudy has been deposited in the National Omics Datautational Biology, CAS-MPG Partner Institute for Computational Biology, SIBS, CAS, Shanghai, 200031 China; 3 Skolkovo Institute of Science and Technology, Moscow, 143028 Russia; 4 ShanghaiTech University, Shanghai, 200031 China; 5 Department of Psychiatry and Friedman Brain Institute, Icahn School of Medicine at Mount Sinai, New York, NY 10029 USA; 6 Max Planck Institute for Molecular Plant Physiology, Am Mühlenberg 1, Potsdam, 14476 Germany; 7 Max Planck Institute for Evolutionary Anthropology, Leipzig, 04103 Germany; 8 Comparative Biology Group, CAS-MPG Partner Institute for Computational Biology, SIBS, CAS, Shanghai, 200031 China

**Keywords:** Psychiatric disorders, Molecular biology

## Abstract

Lipids are essential to brain functions, yet they remain largely unexplored. Here we investigated the lipidome composition of prefrontal cortex gray matter in 396 cognitively healthy individuals with ages spanning 100 years, as well as 67 adult individuals diagnosed with autism (ASD), schizophrenia (SZ), and Down syndrome (DS). Of the 5024 detected lipids, 95% showed significant age-dependent concentration differences clustering into four temporal stages, and resulting in a gradual increase in membrane fluidity in individuals ranging from newborn to nonagenarian. Aging affects 14% of the brain lipidome with late-life changes starting predominantly at 50–55 years of age—a period of general metabolic transition. All three diseases alter the brain lipidome composition, leading—among other things—to a concentration decrease in glycerophospholipid metabolism and endocannabinoid signaling pathways. Lipid concentration decreases in SZ were further linked to genetic variants associated with disease, indicating the relevance of the lipidome changes to disease progression.

## Introduction

Lipids take up over half of the brain’s dry weight and are known to play important roles as the brain’s main structural components, as well as energy and signaling molecules [1, 2]. While brain organization and function experience drastic changes during development and aging [3–6], the extent and nature of lipid concentration changes accompanying these processes have not yet been well investigated. Previous studies, employing biochemical methods targeted to specific lipid classes [7–16], as well as untargeted mass spectrometry [17], have reported extensive age-dependent changes in the lipid concentration in the human brain. These studies, however, focused on specific lipid classes and had limited sample and age representation. Alterations of lipid compositions were reported to be associated with cognitive disorders, including autism (ASD), schizophrenia (SZ), and Down syndrome (DS), by studies examining lipid concentrations in the blood [18–25] and brain [26, 27].

Here, we investigated lipid concentration levels in the gray matter of the dorsolateral prefrontal cortex (PFC) in 396 cognitively healthy humans with ages spanning 100 years of the human lifespan. Furthermore, we compared age-dependent lipidome changes with the lipidome alterations in disorders that commonly affect cognition, by measuring lipid concentrations in PFC samples of SZ, ASD, and DS patients. Based on the concentration levels of more than 5000 hydrophobic compounds (lipids), we were able to characterize the main features of the lipidome changes across the lifespan with unprecedented temporal resolution, assess the extent of metabolic breakdown in aging and gain insights into the role of lipidome alterations in the three common cognitive disorders.

## Materials and methods

### Samples

Samples from cognitively unaffected human controls were collected from the NICHD Brain and Tissue Bank for Developmental Disorders and the Maryland Psychiatric Research Center at the University of Maryland, the Maryland Brain Collection Center, the Netherlands Brain Bank, and the Chinese Brain Bank Center (CBBC, http://cbbc.scuec.edu.cn, Wuhan, China). Samples from patients with DS were collected from the Netherlands Brain Bank. Samples from patients with ASD or SZ were collected from NICHD Brain and Tissue Bank for Developmental Disorders at the University of Maryland, the Harvard Brain Tissue Resource Center, and the Maryland Psychiatric Research Center. Brain tissue from subjects with ASD was obtained through the Autism Tissue Program [28]. Diagnosis of SZ is based on DSM IV-based criteria and was provided by the participating brain banks. Written consents for the use of human tissues for research were obtained either from the donors or their next-of-kin. More detailed information of human samples is provided in Table S1. According to the protocol of the CBBC, specific permission for brain autopsy and use of the brain tissue for research purposes was given by the donors or their relatives. All tissue samples were shipped by the brain banks without accompanying personal identifier information.

All human samples in this project were extracted from the PFC, which were dissected from the anterior part of the superior frontal gyrus. The sample weights are 12.55 ± 1.65 mg. All samples were well-preserved postmortem samples that had been stored at −80 °C before RNA or lipid extraction. To provide sufficient temporal resolution, the number of cognitively healthy individuals sampled in our study was substantially larger than in previous studies [12, 17]. The numbers of ASD or SZ patients were comparable to the ones sampled in previous studies [19, 22, 23].

### MS sample preparation and measurements

Metabolites were extracted from 10–15 mg of frozen tissue, which was homogenized by a ball mill to a fine powder, as described elsewhere [29]. In brief, the frozen tissue was transferred to cooled 2 ml round-bottom microcentrifuge tubes and each sample was re-suspended in 1 ml of a −20 °C methanol:methyl-tert butyl-ether (1:3 (v/v)) mixture, containing 1.5 μg of 1,2-diheptadecanoyl-sn-glycero-3-phosphocholine (Avanti Polar Lipids, 850360P). The samples were immediately vortexed before they were incubated for 10 min at 4 °C on an orbital shaker. This step was followed by ultra-sonication in an ice-cooled bath-type sonicator for additional 10 min. To separate the organic from the aqueous phase 650 μl of a H_2_O:methanol mix (3:1(v/v)) was added to the homogenate, which was shortly vortexed before it was centrifuged for 5 min at 14,000×*g*. Finally, 500 μl of the upper MTBE phase, which contains the hydrophobic compounds (lipids), was sampled to a fresh 1.5 ml microcentrifuge tube. This aliquot can either be stored at −20 °C for some weeks or immediately concentrated to complete dryness in a speed vacuum concentrator at room temperature.

Prior to analysis, the dried pellets were re-suspended in 400 μl acetonitrile:isopropanol (7:3 (v:v)), ultra-sonicated and centrifuged for 5 min at 14.000×*g*. The cleared supernatant was transferred to fresh glass vials and 2 μl of each sample was injected onto a C_8_ reverse-phase column (100 mm × 2.1 mm × 1.7 μm particles, Waters) using a UPLC system (Acquity, Waters, Manchester, UK). In addition to the individual samples, we prepared pooled samples, namely 10 µl of each sample was mixed. These pooled samples were measured after every 20th sample, providing us information on system performance including information on sensitivity, retention time consistency, sample reproducibility, and compound stability.

The mobile phase for the chromatographic separation consisted of 1% 1 M NH_4_acetate and 0.1% acetic acid in UPLC MS grade water (Buffer A, BioSolve, Valkenswaard, Netherlands), while Buffer B contained 1% 1 M NH_4_acetate and 0.1% acetic acid in acetonitrile/isopropanol (7:3 (v:v), BioSolve). The flow rate of the UPLC system was set to 400 μl/min. The gradient was 1 min isocratic flow at 45% A, 3 min linear gradient from 45 to 25% A, 8 min linear gradient from 25 to 11% A, and 3 min linear gradient from 11 to 1% A. After cleaning the column for 4.5 min at 1% A, the buffer was set back to 45% A, and the column was re-equilibrated for 4.5 min, resulting in a final run-time of 24 min per sample.

The mass spectra were acquired using an Orbitrap-type mass spectrometer (Orbitrap-XL, Thermo-Fisher, Bremen, Germany). The spectra were recorded using full-scan mode, covering a mass range from 100 to 1500 *m*/*z*. The resolution was set to 60,000 with 2 scans per second, restricting the maximum loading time to 100 ms. The samples were injected using the heated electrospray ionization source (HESI), and the capillary voltage was set to 3.5 kV in positive and negative ionization mode. The sheath gas flow value was set to 40, while the auxiliary gas flow was set to 20. The capillary temperature was set to 200 °C, while the drying gas in the heated electrospray source was set to 350 °C. The skimmer voltage was set to 20 V, while the tube lens was set to a value of 140 V. The spectra were recorded from 0 to 20 min of the UPLC gradients. Sample randomization was performed twice: before the lipid extraction and before the mass spectrometry measurements. Persons performing lipid extraction and lipidome measurement were unaware of sample information, including age and diagnostic status. The correlation between the measurement order and sample age were not significant (positive ionization mode: *ρ* = 0.006, *P* > 0.8; negative ionization mode: *ρ* = −0.002, *P* > 0.9). Similarly, there was no significant relationship between measurement order and health status or ethnicity (Wilcoxon rank-sum test, nominal *P* > 0.3). The MS preparation and measurement procedure for autistic samples in DS2 has been described elsewhere [17].

### Lipidome data preprocessing

MS lipids were extracted and aligned across samples using Progenesis QI software (Version 2.3, Nonlinear Dynamics, Newcastle upon Tyne, UK) according to the vendor description. Only the reliably detected lipids satisfying the following criteria were retained: (a) retention time (RT) ≥0.6 min; (b) detected in at least 90% of pooled samples and at least 80% of non-pooled samples. Quantile normalization was applied to the log10-transformed concentration of reliably detected lipids. The batch effect correction was done based on a linear regression model.

Data preprocessing procedure for lipid concentrations measured in DS2 has been described elsewhere [17]. The batch effect correction was done using the fitted support vector regression model with a Gaussian kernel, considering the concentration of each lipid as a function of the measuring order of the samples. An upper quartile normalization was used to normalize the lipid concentration measurements.

### RNA-seq dataset

72 postmortem PFC samples were selected from the 72 individuals used for lipidome measurements, with square-root- transformed ages uniformly distributed along the whole lifespan. Of them, 19 individuals were measured previously [30]. Sample weights are 20–30 mg. RNA integrity numbers (RIN) of 62 out of the 72 samples were not lower than six. For the other ten samples, although their RINs were lower than six or unmeasurable, their 28S/18S rRNA ratios were around two, supporting that these samples were suitable for RNA-seq measurement. More detailed information of human samples is provided in Table S1. The poly(A) + RNA enriched cDNA library was constructed using the Illumina TruSeq® standard mRNA sample preparation kit. The RNA fraction was sequenced on the Illumina HiSeq 4000 platform in pair-ended mode, each mate in length of 150 nt. The sample order was randomized prior to RNA extraction and sequencing. The person performing RNA extraction, cDNA library construction, and RNA sequencing were unaware of sample information, including age and ethnicity.

### RNA-seq data preprocessing

The paired-ended RNA-seq reads were trimmed with Fastx to remove adapter sequences. Only read pairs with lengths of at least 75 nt for both mates after trimming were kept. The trimmed reads were mapped to the human genome hg38 with in-build SNPdb information using HISAT2 [31] with default parameters. Read counting was done using HTseq [32] for protein-coding and lincRNA genes annotated in GENCODE version 24. DESeq2 [33] was used for gene expression normalization and FPKM (fragments per kilobase per million reads) was used to represent the gene expression level of each gene in each sample. Genes with FPKM > 1 in at least one sample (expressed genes) were used for a downstream analysis.

### Human lipidome stage classification and stage-dependent lipid identification

To classify the stages in human lipidome compositions, we used a sliding window-based procedure. At each step, samples were selected using the sliding window across samples in ascending order of ages. The window size was set to be 10 samples, with step size set to be five samples. For each lipid, a Wilcoxon rank-sum test was applied to each lipid comparing its concentrations in the selected samples with those in the other samples. Lipids with nominal *P* < 0.01 in at least one window were defined as window-dependent lipids. Numbers of window-dependent lipids in all windows were summed up to get the window-dependence identification frequency. Permutations of sample ages were performed 100 times. The *P* value of the window-dependent lipid identification was calculated as a proportion of cases in which frequencies in permutations are larger than or equal to those obtained from actual data. A lipid concentration specificity matrix of all window-dependent lipids with *N* rows and *M* columns, denoted as **D**, was generated, where *N* was the number of lipids, and *M* was the number of sliding windows. *D*_*ij*_ was set to 1, 0, or −1, each of which represented the specificity of the *i*-th lipid in the *j*-th window (1 means significantly higher concentrations in samples in the window, −1 means significantly lower concentrations in samples in the window, and 0 means no significant difference). Hierarchical clustering was then applied to the sliding windows, with distance between each pair of windows calculated as 1−*ρ*, where *ρ* is the Spearman’s correlation coefficient between two columns in **D** representing two windows. A visual inspection of the dendrogram structure was used to determine the number of stages. To classify stages in transcriptome data and DS2 lipidome data, we used similar approaches with the following modifications: window size = 4 samples; step size = 2 samples; lipids/genes with nominal *P* < 0.05 in at least one window were defined as window-dependent lipids/genes.

To identify the stage-dependent lipids in DS1 and DS2 human data, we applied the Wilcoxon rank-sum test to each window-dependent lipid for each stage, comparing its concentrations in samples within the stage with the other samples. Lipids with Benjamini and Hochberg (BH) corrected *P* < 0.05 were defined as stage-dependent of the stage. To link lipids in DS1 and DS2 human data, we required lipids to have only one correspondence in the other dataset with *m*/*z* difference <10 ppm and share at least one LIPID MAPS annotation.

Each stage-dependent lipid was assigned to at least one of the eight stage-dependent groups according to its significance and changing direction at each stage. The igraph package was used to visualize concentration correlations of lipids within each group. For each of the three groups, namely S1-L, S2-L, and S4-H, two sub-groups were identified with hierarchical clustering using 1−*r* as distance, where *r* represents the pairwise Pearson’s correlation coefficients of lipid concentrations across all cognitively healthy samples in DS1.

### Lipid annotation and enrichment analysis

Lipid annotations were performed using mass search with a tolerance of 10 ppm against the LIPID MAPS database annotation, using the list of adducts described elsewhere [29]. Lipid classes were assigned to the annotated lipids according to the LIPID MAPS database classification. Pathways were assigned according to the KEGG database annotation. Overrepresentations of lipid classes and pathways compared to random sampling from detected lipids with annotations were tested using one-sided Fisher’s exact tests and hypergeometric tests, respectively, followed by BH corrections. Significant enrichment was defined as BH-corrected *P* < 0.05 and *P* < 0.1.

### Lipid-interacting gene support

Expressed genes with direct interaction with reliably detected lipids according to KEGG annotations were used to calculate correlations between temporal profiles of lipids and genes. A Spearman correlations coefficient (*ρ*) between gene expression levels and lipid concentrations across samples were calculated in age sorted and age permutated data. Permutation of sample ages was performed 100 times. *P* was defined as the proportion of cases in which median absolute *ρ* in age-permuted data was larger than or equal to that obtained in age sorted data. For stage-dependent lipids, all 72 samples with RNA-seq data were used, while for aging-related lipids, only samples in the adult stage were used.

To quantitatively assess the proportion of the lipidome variation along the lifespan explained by the expression changes of the interacting genes, we conducted a LASSO-constrained multivariate linear modeling based analysis. This method is analogous to the methods previously used to estimate the proportion of variations of gene expression differences explained by the differential binding of transcription factors (TFs) [34]. Specifically, we constructed a model that predicts the concentration difference between lipid concentration in a sample and the average concentration of this lipid over the lifespan (scaled lipid concentrations) based on the scaled expression levels of lipid-interacting genes. Scaled data was defined as

$$d_{ij}\prime = \frac{{d_{ij} - \mu }}{\sigma }$$

where *d*_*ij*_ represents log10-transformed data of the *i*th lipid or genes in the *j*th sample, *μ* and *σ* represents average and standard deviation of the *i*th lipid or gene across all 72 samples, respectively.

For each of the 677 stage-dependent lipids linked to genes in the KEGG annotation, we constructed an independent model using the concentration of this lipid as a response variable and expression levels of 936 lipid-interacting genes annotated in KEGG, as potential explanatory variables. The model was constructed based on scaled lipid concentration and gene expression values in 71 samples and applied to predict scaled lipid concentration (i.e. the difference between the concentration of this lipid in the sample and the average concentration of this lipid across the lifespan) in the remaining sample. The explanatory power of the scaled gene expression values was quantified as squared Pearson correlation coefficients between the predicted scaled lipid concentrations within a sample and the actual concentrations.

### Quantification of effects of different factors on lipidome concentration variation

To quantify the proportion of variance of lipidome explained by age, we fit the following formula for each lipid:

$$Y_{ij}\, = \beta _{0i} + \beta _{1i}A_{j} + \beta _{2i}A_j^{2} + \beta _{3i}A_j^{3} + \varepsilon _{ij},$$

where *Y*_*ij*_is the lipid concentration for lipid *i* and sample *j*, *A*_*j*_ is the age of the sample *j* in square-root scale and *ε*_*ij*_ is the error term. For each lipid, the age effect on its concentrations was quantified as the ratio of the explained sum of squares (ESS) relative to total sum of squares (TSS). To get the average concentration variance explained by age, ESS and TSS for all lipids were summarized, respectively, and then their ratios were calculated. Permutations of ages 100 times were used to estimate the significance of variance explained by age. A similar procedure was also applied to quantify the variance explained by each of the other factors including PMI, ethnicity, RIN, and sex, while linear models instead of the cubic polynomial model was used.

### Change magnitude quantification

To calculate the change magnitude of lipid concentration along lifespan, we fit a natural spline interpolation model (implemented as smooth.spline function in R) to each lipid, with a variable degree of freedom from two to eight, using lipid concentration as the response variable and the square-rooted ages as the predictor variable. The degree of freedom was determined for each lipid separately, based on the adjusted *r*^2^ criterion. Based on the interpolation model of each lipid, its concentration at 19 uniformly distributed age points along the lifespan were interpolated. The absolute concentration differences between neighboring age points were then calculated and normalized to the median concentration of the lipid across all the interpolated age points. The median of concentration differences across all the lipids was then calculated as the average lipidome changing magnitude for each of the 18 age intervals. The average lipidome change magnitudes of all age intervals were summed up to get the total change magnitude. Permutations of sample ages were performed 1000 times to estimate the random background. Permutation *P* was calculated as the proportion of cases in which total change magnitude in a permutation was larger than or equal to that in actual data. For a quantification of change magnitude in adult stage, a similar procedure was applied with some modifications: only cognitively healthy samples not younger than 30 years of age were used; and lipid concentrations at 15 evenly distributed age points in the adult stage were interpolated; age was in the linear scale.

### Identification of lipids with aging-related concentration profile in adult stage

To identify lipids with aging-related concentration profiles in the adult stage, we tested the temporal dynamics of the concentration profile of each lipid by using the polynomial regression model with sample ages as the predictor variable and employing the F-test [35]. Lipids with F-test *P* *<* 0.05 after BH correction were considered as with age-related concentration profiles in adults (referred as aging-related lipids).

### Identification of transition points of concentration profiles

To identify transition points of lipid concentration profiles in the adult stage, we used a previously described procedure [36]. Briefly, the concentrations of each aging-related lipid were interpolated based on the natural spline interpolation model (*df* = 5) at 27 uniformly distributed age points along the adult stage. Using each of the 25 internal age points as the potential transition point, F-test was employed to compare the piecewise linear model and the non-piecewise counterpart, with the most likely transition point determined as the one resulted in the smallest F-test *P* value. The *P* value threshold to identify the effective transition point for each lipid was determined based on FDR and *P* value estimated by permutations of ages 100 times, as the largest *P* value cutoff resulted in FDR < 0.8 and *P* < 0.01 in permutation test. We used similar procedure to identify transition points of aging-related lipids in C1 along the whole lifespan with modifications as following: ages were in square-root scale; the *P* value threshold was defined as the largest *P* with FDR < 0.2 and *P* < 0.01 in permutation test.

### Disorder-associated (DA) lipid identification

DA analyses were conducted for patient samples of each neural disorder and matched control samples, respectively. To eliminate the confounding effects of ages, only the cognitively healthy samples with matched age ranges as the patient samples, i.e. 18–65 years old, were used. The Han Chinese cognitively healthy samples were also removed from the analysis to avoid confounding the effect of ethnicities. Two latent batches in the dataset were detected by hierarchical clustering, based on the pairwise distance between each pair of samples defined as 1−*r*, where *r* is the Pearson’s correlation coefficient between the two samples across concentrations of all the detected lipids. The latent batch effect on the lipidome was corrected using the linear model as that for the batch effect correction.

Based on the corrected lipid concentrations, the DA lipids were identified using Wilcoxon rank-sum test to compare the lipid concentrations in patient samples of each disorder and the control samples, with nominal *P* *<* 0.01. Permutation of diagnostic status 1000 times was used to estimate test significance (*P* value).

For ASD DS2, we used feature selection based on logistic regression with L1 regularization to identify lipids with differential concentration between ASD and control. Random division of test set and training set was performed 500 times. Top 600 lipids with the highest probability to be chosen in the model were defined as ASD-associated lipids. Pathway enrichment of these ASD-associated lipids was conducted using hypergeometric test.

### Quantification of disorder effect on lipid concentration variance

To estimate the lipid concentration variance explained by the diagnostic status, we fit a linear model for each lipid, using lipid concentration as the response variable and diagnostic status as the predictor variable. The disorder effect was quantified as the ratio of the ESS relative to the TSS. To perform estimations with age, sex, PMI distributions, and sample size equalized among disorders, we performed bootstrapping 100 times (sample size = 5) and fit two models to each detected lipid. The null model considered the effect of age, sex, and PMI on lipid concentration, while the full model further considered the effect of the disorder. The proportion of variance explained by diagnostic status was calculated as the difference of the residual of null model and that of the full model relative to the residual in the null model.

### Data availability

Primary RNA sequence data of this study has been deposited in the National Omics Data Encyclopedia (NODE, http://www.biosino.org/node/index) database with project ID NODEP00371662. The processed gene expression data, processed lipid concentration data for lipids in DS1 samples, as well as DS2 control and ASD samples, and lipid annotation information for lipids in DS1 and DS2 are available at https://data.mendeley.com/datasets/m4dt3z68s5/draft?a=85342504-8750-4703-88bc-83bf0c111935 with reserved 10.17632/m4dt3z68s5.1.

### Code availability

The source codes for the temporal stage classification and transition point identification are available at https://data.mendeley.com/datasets/m4dt3z68s5/draft?a=85342504-8750-4703-88bc-83bf0c111935 with reserved 10.17632/m4dt3z68s5.1.

## Results

### PFC lipidome undergoes substantial changes throughout the human lifespan

We measured concentrations of hydrophobic compounds with molecular weights below 1500 Da (lipids) in 403 human postmortem PFC samples from 396 cognitively healthy individuals aged between 18 weeks post conception to 99 years (Table S1 and Figure S1). The untargeted analysis conducted using liquid chromatography coupled with mass spectrometry (LC-MS) in both positive and negative ionization modes yielded concentration estimates for a total of 5024 lipids, 2222 of them annotated using LIPID MAPS database.

**Fig. 1:**
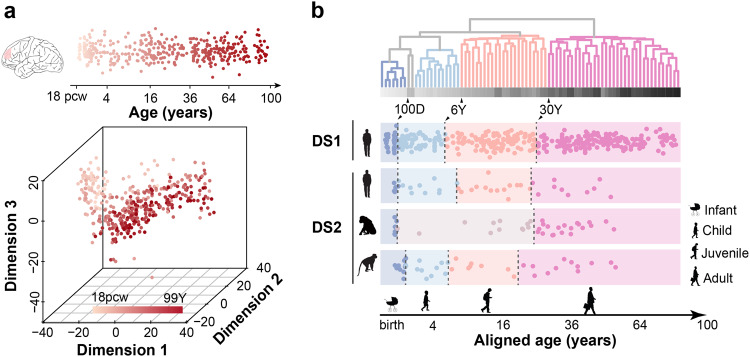
Lifespan stages of the human PFC lipidome. **a** Upper panel: age distribution of the 396 cognitively healthy individuals used in the study. The dissection area is marked in pink. Lower panel: total lipidome variation among 403 samples from the 396 individuals visualized using multi-dimensional scaling (MDS). The MDS plot shows Euclidean distances calculated using concentrations of 5024 detected lipids. Each dot represents a sample. The colors represent individuals’ ages with darker shades corresponding to older individuals. **b** Upper panel: the dendrogram of the 79 sliding windows covering the lifespan. The branch length shows (1−*ρ*) distances, where *ρ* is the Spearman correlation coefficient of lipid concentration specificity measures for 4777 lipids with differential concentration in at least one sliding window. The branch color shows lifespan stages: blue—infant; light blue—child; orange—juvenile; pink—adult. The horizontal gray bar indicates the median age in each window, with darker shades corresponding to older age. Numbers show boundary ages (D—days, Y—years). Lower panel: lifespan stage boundaries in the DS1 and public human, chimpanzee, and macaque data (DS2). Silhouette symbols indicate species and lifespan stages. Each dot represents a sample. The x-axis shows square-root-transformed age of assessed individuals. Age of non-human primates was corrected for differences in species’ maximal longevity. The background color represents lifespan stages as in the dendrogram above, except that light brown indicates the combined child and juvenile stage in the chimpanzee

A visualization of lipid concentration variations among samples revealed clear temporal differences (Fig. 1a). Quantitatively, age explained by far the largest proportion of the total lipidome variation (19.56%, permutations, *P* < 0.01), compared to the other factors, such as sex (0.27%, permutations, *P* = 0.3), ethnicity (3.69%, permutations, *P* < 0.01), postmortem interval (PMI) (0.67%, permutations, *P* = 0.06) and sample quality, estimated based on RNA preservation (0.69%, permutations, *P* = 0.14).

### The lifelong PFC lipidome changes group into four stages

To characterize age-dependent lipidome changes, we identified lipids with concentration levels particular to a certain age interval using a sliding window approach. Specifically, we searched for lipids with concentrations in a given temporal window different from the rest of lifespan (Wilcoxon rank-sum test, nominal *P* < 0.01). Each window contains ten samples in ascending age order. More than 95% (4777) of all detected lipids satisfied this criterion in at least one window (permutations, *P* < 0.1).

An unsupervised clustering of the concentration specificity profiles revealed four temporal stages: (i) infant stage ending at approximately 100 postnatal days (90% confidence interval [CI]: 10–500 days); (ii) child stage ending at approximately 6 years (90% CI: 4–14 years); (iii) juvenile stage ending at approximately 30 years (90% CI: 16.5–41.5 years); and (iv) adult stage extending from 30 years on (Fig. 1b). The separation of the PFC lipidome into four stages was not caused by confounding factors, such as sex, ethnicity, PMI or sample preservation (Figure S1). Furthermore, an independently generated human PFC lipidome dataset [17] showed consistent lipidome stage separation (Fig. 1b and Figure S2). Notably, chimpanzee and macaque PFC lipidome data (number of individuals *N* = 40 for chimpanzees and *N* = 40 for macaques) [17] also revealed the same lipidome stages after correction for differences in species’ maximal lifespan (Fig. 1b and Figure S2).

To assess the relationship between age-dependent lipidome changes and changes in the expression of genes encoding proteins directly interacting with lipids according to KEGG annotation (lipid-interacting genes), we measured transcriptomes in 72 of the 396 individuals used for the lipidome measurements, retaining the lifespan coverage (Table S1). Based on the generated RNA-seq data, we detected the expression of 16,448 genes covered by a total of 1,285,419,776 pair-end reads. The proportions of data variation explained by individuals’ age, ethnicity, and sex, as well as postmortem delay duration did not differ substantially between the transcriptome and the lipidome data (Figure S1). The same analysis applied to the expression levels of 646 lipid-interacting genes showed that a concordant stage pattern also exists at the transcriptome level (Figure S3).

### Distinct properties of stage-dependent lipid groups

**Fig. 2:**
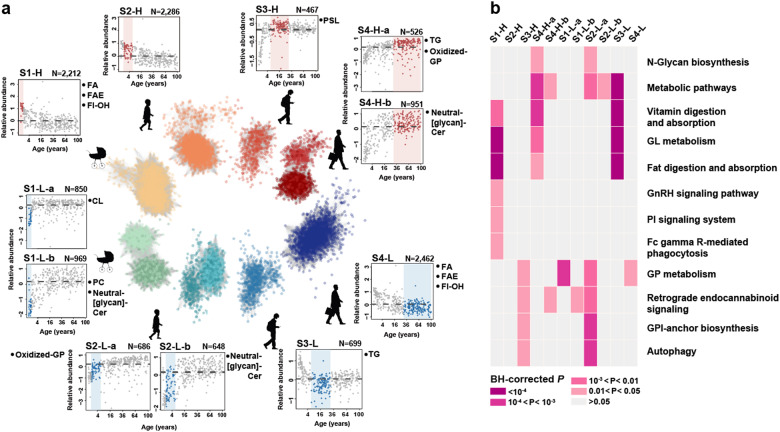
Characteristics of stage-dependent lipids. **a** Average temporal profiles and lipid class enrichment of eleven lipid groups. The lipid groups represent network modules based on concentration profiles of stage-dependent lipids across 403 samples from 396 individuals. Each dot represents a lipid. Silhouette symbols indicate lifespan stages. The panels show the average concentration patterns in each group, where each dot represents one sample, and group names and lipid numbers are shown on top. Dash lines show the median relative lipid concentration within a group. Colored areas and dots indicate lifespan stages with differential lipid concentrations (red—higher concentration; blue—lower concentration). Abbreviations next to the panels represent enriched lipid classes (FA fatty acids and conjugates, FAE fatty esters, Fl-OH flavonoids, CL glycerophosphoglycerophosphoglycerols (cardiolipins), PC glycerophosphocholines, Neutral-[glycan]-Cer neutral glycosphingolipids, Oxidized-GP oxidized glycerophospholipids, PSL phosphosphingolipids, TG triradylglycerols). **b** KEGG pathways enriched in stage-dependent lipids. The heatmap shows the BH-corrected *P* values of the hypergeometric tests

As many as 4031, 3620, 1166, and 3939 lipids displayed distinct concentration levels at the infant, child, juvenile, and adult stages, respectively (stage-dependent lipids, Fig. 2, Wilcoxon rank-sum test, BH-corrected *P* < 0.05). Stage-dependent concentration differences agreed well between the current and the published datasets [17] based on the analysis of 219 unambiguously linked lipids defined as stage-dependent in both datasets (random sampling, *P* < 0.01; Figure S2). Furthermore, the concentrations of stage-dependent lipids correlated significantly with the expression of the corresponding lipid-interacting genes (median absolute Spearman correlation coefficient *ρ* = 0.12; permutations, *P* < 0.01; Figure S3).

Considering that lipid concentration might be influenced by multiple genes, we quantified the proportion of the temporal lipidome variation explained by the expression of multiple lipid-interacting genes using LASSO-constrained multivariate linear modeling. This approach is analogous to the methods previously used to estimate the proportion of variation of gene expression differences explained by the differential binding of transcription factors (TFs) [34]. Specifically, we constructed a model that predicts the concentration difference between lipid concentration in a sample and the average concentration of this lipid over the lifespan (scaled lipid concentrations) based on the scaled expression levels of lipid-interacting genes. The results demonstrate that for samples in the infant and child stages, where lipid concentrations differ substantially from the average lifespan concentrations, gene expression difference-based predictions explained on average 49% and 14% of the lipid concentration differences, respectively (median values). By contrast, for samples in the juvenile and adult stages, which showed fewer lipid concentration differences compared to the average concentration across the lifespan, the predictive accuracy was expectedly lower, explaining on average 3.5% and 5.5% of the lipid concentration differences, respectively. Notably, the proportion of variation of lipid concentration differences explained by the differential expression of lipid-interacting genes in infant samples was comparable to the reported proportions of variation of gene expression differences explained by differential TF binding estimated using a similar approach: *r*^*2*^ = 53% [34].

The separation of stage-dependent lipids according to the direction of the concentration change, followed by clustering based on Pearson’s correlations between concentration profiles across the lifespan, revealed 11 lipid groups (Fig. 2a). Among them, 10 were enriched in nine specific lipid classes (one-sided Fisher’s exact test, BH-corrected *P* < 0.05; Fig. 2a and Table S2), and 12 functional pathways (hypergeometric test, BH-corrected *P* < 0.05; Fig. 2b and Table S2). The pathways included “retrograde endocannabinoid signaling” (also known as endocannabinoid (eCB) signaling) overrepresented in lipid groups with low concentrations at infant and child stages, and elevated at juvenile and adult stages, as well as “phosphatidylinositol (PI) signaling system” characterized by the elevated lipid concentrations at the infant stage.

### PFC lipidome alterations at advanced age

**Fig. 3:**
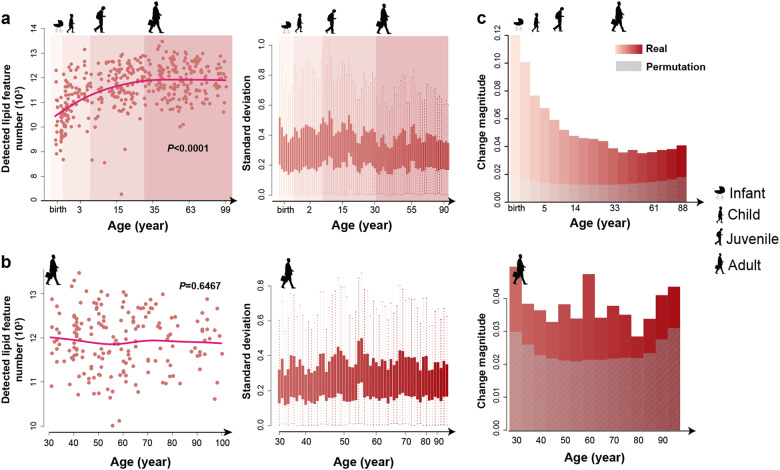
Complexity and variability of the PFC lipidome. **a** Left panel: number of lipids detected in each sample across the lifespan, with four stages indicated by colors and silhouette symbols. Lines represent spline curves built with three degrees of freedom. Right panel: standard deviation distributions of log10-transformed concentrations of 5024 lipids within a sliding window (window size = 10 samples, step size = 5 samples). Boxes show the first and third quartiles of the distribution. Error bars show the maximum and minimum standard deviation values. **b** Number of detected lipids and standard deviations of the lipid concentrations at the adult stage (window size = 6 samples, step size = 3 samples). **c** Median magnitude of concentration differences of 5024 detected lipids within 18 age intervals evenly spaced across the lifespan (red bars, top) and 14 intervals evenly spaced across the adult stage (red bars, bottom). The magnitude of concentration differences for each lipid was calculated as the ratio of lipid concentration difference within an age interval and the median lipid concentration across intervals. The gray bars show the median magnitude of lipidome differences expected by chance based on permutations of the age labels 1000 times. The x-axis labels show the median age of each interval

Some of the proposed mechanisms of aging predict increases in numbers of detected compounds at advanced age due to metabolic dysfunction and other age-related deregulation [37–39]. We detected 10,185 ± 791, 11,203 ± 794, 11,637 ± 909, and 11,912 ± 662 lipids at infant, child, juvenile, and adult stages, indicating a progressive increase in the lipidome complexity of human PFC from infants to adults (ANOVA, *P* < 0.0001; Fig. 3a). There was, however, no significant increase of detected lipid numbers at the adult stage from age 30 onwards to 99 years, strongly implicating that complexity plateaus upon completion of the extended period of PFC development (ANOVA, *P* = 0.6467; Fig. 3b). Furthermore, variance of the lipid concentration levels did not increase at advanced age (Fig. 3b).

**Fig. 4:**
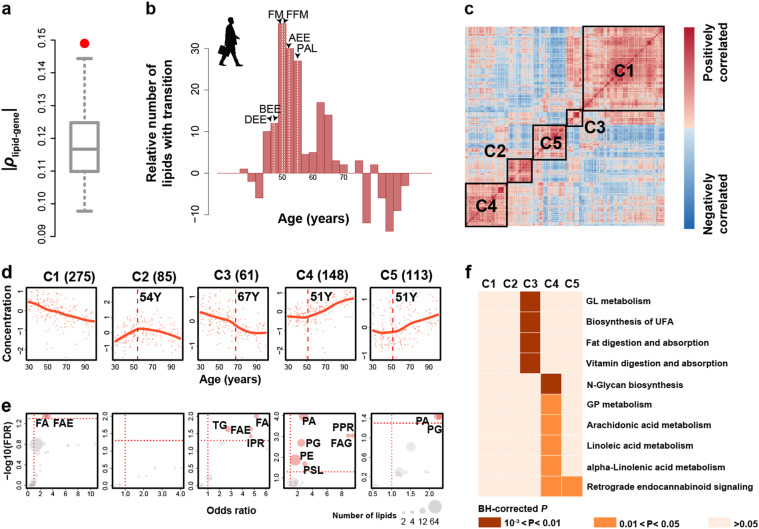
Characteristics of aging-related lipidome changes. **a** Correlation between concentrations of aging-related lipids and expression of their interacting genes during the adult stage. The red dot shows the median absolute Spearman correlation coefficients (*ρ*). The gray box shows the background distribution of absolute *ρ* medians calculated by permutations of age labels 100 times. **b** Age distribution of transition points of the lipid concentration profiles during the adult stage. The y-axis shows the number of transition within a given age interval minus the background calculated by permutations of age labels 100 times. Light yellow dash lines show the age of transition points for fat mass (FM), fat-free mass (FFM), physical activity level (PAL), daily (DEE), basal (BEE) and activity energy expenditure (AEE) measurements from 529 individuals [58]. **c** The heatmap based on (1−*ρ*) distances among 682 aging-related lipids calculated using their concentrations across 178 samples from 174 adult individuals. Black boxes and labels show five clusters. **d** Temporal patterns of lipid concentrations in the five clusters. Cluster names and lipid numbers are shown on top. Each dot represents the average relative lipid concentration within a sample. The lines represent spline curves built with five degrees of freedom. The vertical dash lines show the modes of the transition point distribution. **e** Lipid classes enriched in the five clusters. Each dot represents the enrichment of a lipid class, with the size proportional to the number of overlapping lipids (red—significant enrichment; gray—not significant). The vertical dash lines indicate *OR* = 1. The horizontal dash lines indicate BH-corrected *P* = 0.05 of one-sided Fisher’s exact test. IPR isoprenoids, PPR polyprenols, PG glycerophosphoglycerols, PA glycerophosphates. **f** KEGG pathways enriched in the five clusters. Colors show the BH-corrected *P* values of hypergeometric tests

Yet, even at the adult stage lipidome continues to change quantitatively (permutations, *P* < 0.001; Fig. 3c), with a total of 682 lipids showing significant concentration differences between 30 and 99 years of age (aging-related lipids) (ANCOVA, BH-corrected *P* < 0.05). Supporting the authenticity of this observation, the concentration levels of the aging-related lipids correlated significantly with the expression of their interacting genes within the adult stage interval (permutations, *P* = 0.01; Fig. 4a).

To further assess when lipidome changes characteristic of the last decades of life start, we determined the transition points of the lipid concentration profiles within the adult stage. Of the 682 lipids, 523 (77%) had a detectable concentration profile transition point (permutations, *P* < 0.01). The transition point locations formed a bimodal distribution with the major peak located at 50–55 years, and the minor peak at 65–70 years of age (Fig. 4b).

A hierarchical clustering of aging-related lipids resulted in five clusters (Fig. 4c). The 50–55-year transition point was apparent in clusters C2, C4, and C5, and the 65–70 transition point in cluster C3 (Fig. 4d). The profile of the largest cluster C1 (*N* = 275, 40.3% of all aging-related lipids) represented a steady concentration decrease over the entire span of the adult stage. An extending analysis of C1 concentration profiles to earlier life stages identified the presence of earlier additional transition points at 2 and 31 years of age (Figure S4). Lipids in clusters C1, C3, C4, and C5 were enriched in specific lipid classes and functional pathways (Fisher’s exact test, BH-corrected *P* < 0.05; Fig. 4e, f, Table S3), such as biosynthesis of unsaturated fatty acid in C3, glycerolipid metabolism in C4, as well as retrograde eCB signaling in C4 and C5, suggesting the non-random character of aging-related changes. Notably, a transition point analysis conducted separately for male (*N* = 115) and female (*N* = 63) adult individuals revealed differences in transition point distribution between sexes (Figure S5).

### The PFC lipidome composition is altered in common cognitive disorders

**Fig. 5:**
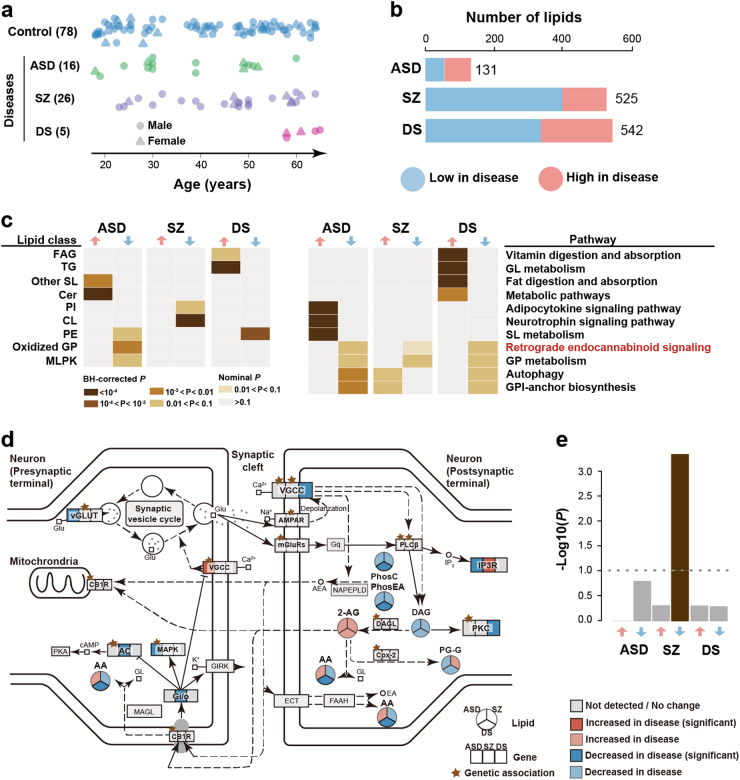
Disorder-associated (DA) lipidome changes in the human PFC. **a** Age distribution of patients with autism (ASD, green), schizophrenia (SZ, purple), Down syndrome (DS, pink), and matched controls (blue). Each symbol represents an individual (circle—males, triangle—females). Numbers in brackets show numbers of individuals in each group. **b** Numbers of DA lipids for each disorder (red—higher concentration in disease, blue—lower concentration in disease). **c** Lipid classes (left) and KEGG pathways (right) enriched in DA lipids. Colors show *P* values of hypergeometric tests. FAG fatty acyl glycosides, MLPK macrolides and lactone polyketides, Cer ceramides, OtherSL other sphingolipids, PI glycerophosphoinositols, PE glycerophosphoethanolamines. **d** Schematic representation of DA genetic, gene expression and lipid concentration changes in retrograde endocannabinoid signaling pathway based on the KEGG annotation. Stars mark genes containing genetic variants linked to corresponding disease. Genes with SZ and ASD related genetic variants were retrieved from GRASP (*P* < 0.05) and SFARI respectively. Gene expression level changes were calculated using public data retrieved from GEO (ASD: GSE28521, SZ: GSE53978, DS: GSE5390). **e** Enrichment of DA genetic variants in genes linked to DA lipids. The y-axis shows the –log10-transformed *P* value of the hypergeometric test

To further assess potential functionality of lipidome composition differences among the lifespan stages, we analyzed lipidome changes in common cognitive disorders: ASD, SZ, and DS. Specifically, we measured the lipidome composition in PFC samples from 26 SZ, 16 ASD and five DS patients aged 18 to 65 (Fig. 5a and Table S1). The disorder samples were measured together with 403 samples from 396 cognitively healthy individuals in a random order (Table S1).

For the 18–65 age interval, the diagnostic status explained the largest proportion of the total lipid concentration variation (6%, permutations, *P* < 0.01), compared to age (4%, permutations, *P* < 0.01) and PMI (1.7%, permutations, *P* < 0.01). Other factors, i.e. ethnicity, sample quality, and sex did not affect lipidome composition significantly (permutations, *P* > 0.15). The effect of the diagnostic status was robust to correction for sample size, age, sex, and PMI for all the three disorders (Wilcoxon rank-sum test, Bonferroni-corrected *P* < 0.0001; Figure S6). Statistically, 10.8%, 10.4%, and 2.6% of detected lipids altered their concentrations significantly in DS, SZ and ASD, respectively (DA lipids; Wilcoxon rank-sum test, nominal *P* < 0.01; *N* = 542 for DS, *N* = 525 for SZ, *N* = 131 for ASD; permutations, *P* < 0.01 for SZ and DS, *P* = 0.06 for ASD; Fig. 5b).

Of the six DA lipid groups, sorted by disorders and concentration change directions, five were enriched in specific lipid classes and all six were enriched in functional pathways (one-sided Fisher’s exact test, BH-corrected *P* < 0.1; Fig. 5c and Table S4). Notably, general lipid concentration decrease in retrograde eCB signaling and “gycerophospholipid (GP) metabolism” pathways was shared among all three disorders. By contrast, pathways enriched in lipids showing increased concentrations in disorders were particular to each disease (Fig. 5c, d).

To assess the validity of these results, we designed a replication experiment and generated an independently measured lipidome dataset containing 33 PFC samples of ASD patients used in [40] and 40 matched controls included in DS2 processed in randomized order (ASD DS2, Table S1). The analysis, based on 9,058 lipids detected in this dataset, yielded consistent pathway enrichment results obtained using all 600 independently identified ASD-associated lipids (one-sided Fisher’s exact test, *P* = 0.02), as well as 319 lipids with decreased concentrations in ASD samples (Pearson correlation coefficient = 0.96, *P* < 0.0001, Figure S7).

An analysis of genetic variants linked to each of the three disorders by genetic and genome-wide association studies and collected in the corresponding databases (GRASP, SZDB, SFARI) revealed strong enrichment in genes linked to lipids with decreased concentrations in SZ (hypergeometric test, *P* = 0.0004; Fig. 5e). This enrichment was robust to the choice of SZ-associated genetic variants from GRASP [41] and SZDB [42] databases (Fig. 5e and Table S5). A functional analysis of these genes yielded 19 pathways containing excesses of SZ-linked genetic variants (hypergeometric test, BH-corrected *P* < 0.01; Table S5). These pathways included PI signaling, which was also identified in lipid class analysis (Fig. 5c), as well as “long-term depression” and “glutamatergic synapse” (Table S5).

### The relationship between age- and disorder-associated lipidome differences

**Fig. 6:**
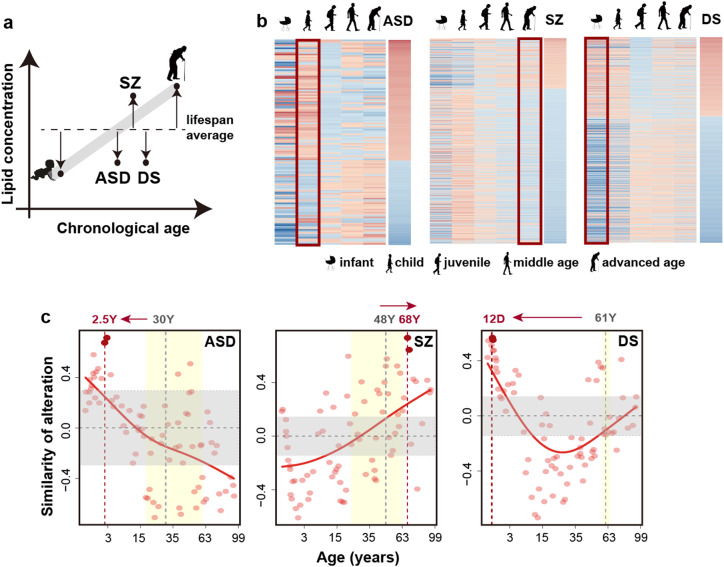
Correlation between disorder-associated (DA) and temporal changes in the human PFC lipidome. **a** Schematic representation of DA and temporal lipidome changes observed in our study. **b** The relationship of DA and temporal lipidome differences among lifespan stages. For each disorder, the left five columns show the relative concentrations of the corresponding DA lipids across lifespan stages (red—higher concentration compared to the lifespan median, blue—lower concentration). The right column shows concentration difference of the corresponding DA lipids between disorder and age-matched control samples (red—higher concentration in disease, blue—lower concentration). The darker shade indicates larger differences. The adult stage is represented by two columns corresponding to 30–65 and 65–99-year intervals. **c** The relationship of DA and temporal lipidome differences among 79 sliding windows covering the lifespan. Dots represent the Pearson correlation coefficient (*r*) values for each window. Curves represent spline curves built with three degrees of freedom. The two windows with the highest PCC values are highlighted in dark red, with red vertical dash lines showing their median age. Yellow shaded areas show patients’ age ranges, with gray vertical dash lines showing the median age. Horizontal gray dash lines and gray shaded areas indicate *r* = 0 and the corresponding 99.8% confidence interval estimated using Rahman *r* distribution [84]

A comparison of DA lipid concentration differences to age-dependent lipidome differences revealed similarity between lipidome alterations in ASD and DS patients with lipidome alterations characteristic for child and infant stages, respectively. By contrast, lipidome alterations in SZ were the closest to the lipidome state at ages greater than 65 years (Fig. 6a, b).

A direct comparison between DA differences and concentration differences particular to a certain age interval identified using a sliding window approach yielded consistent results: lipidome alterations in ASD correlated best with lipidome features particular to 2–4-year-old control individuals, in DS—with lipidome features at the first 50 days of life, and in SZ—with lipidome features of 65–72-year-old control individuals (Fig. 6c). These results were robust with respect to the individual variation (bootstrapping, *P* < 0.001 for ASD, *P* = 0.03 for DS, and *P* = 0.097 for SZ).

### Membrane fluidity alterations inferred based on lipidome composition

The extent of membrane fluidity, an important biological parameter characterizing membrane biochemical and physiological properties, could be inferred from the membrane lipidome composition [2, 43, 44]. We assessed temporal and DA membrane fluidity changes and membrane fluidity alterations in ASD, SZ, and DS, using the following lipidome features: (i) the total cholesterol concentration, (ii) the cholesterol to phospholipids ratio, the proportion of (iii) saturated phospholipids, (iv) glycerophosphocholines (PC), and (v) glycerophosphoethanolamines (PE), which negatively correlating with membrane fluidity, as well as the relative unsaturation state of fatty acyl chains of (vi) GP, (vii) PCs, and (viii) PEs, which positively correlating with membrane fluidity [2, 43, 44].

**Fig. 7:**
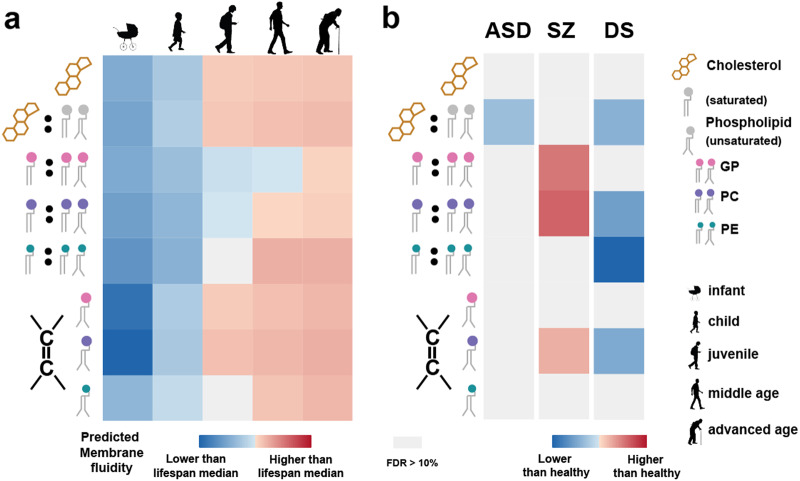
Predicted membrane fluidity levels at lifespan stages and in disorders. **a** Relative membrane fluidity estimates at lifespan stages predicted using the following features (from top to bottom): (i) total cholesterol concentration, (ii) ratio of cholesterol to phospholipid concentration, proportion of saturated (iii) phospholipids, (iv) PC, (v) PE and relative unsaturation degree of fatty acyl of (vi) GP, (vii) PC, and (viii) PE, respectively. Each cell shows relative levels of the features across lifespan stages, with each column representing one stage. The adult stage is shown by two columns representing 30–65-year and 65–99-year intervals. Inverted relative levels are shown for features i–v, which negatively correlate with membrane fluidity. **b** Relative membrane fluidity estimates in three disorders. The features are the same as in panel a. Each cell shows log2-transformed fold change in disorder compared to age-matched controls. Gray cells represent the absence of significant changes (Wilcoxon rank-sum test, BH-corrected *P* > 0.1)

Remarkably, all eight features showed clear increases in membrane fluidity with increased age for all four stages (ANOVA, Bonferroni-corrected *P* < 0.0001), including a further membrane fluidity increase trend at advanced age (65–99 years, Wilcoxon rank-sum test, BH-corrected *P* < 0.1) (Fig. 7a). In disorders, SZ samples demonstrated increased membrane fluidity compared to age-matched controls, while ASD and DS samples had decreased membrane fluidity (Fig. 7b). Notably, these predicted membrane fluidity changes matched well the age effects of the disorders: “older” lipidome state in SZ and “younger” lipidome state in ASD and DS.

Expanding the number of lipidome features associated with membrane fluidity to 26 revealed a more complex picture. Of the 26 features, 24 corresponded to fluidity increase with age. Yet, two features, the length of fatty acyl chain residues in both non-lyso-PCs and non-lyso-PEs, showed the opposite trend (Figure S8).

## Discussion

Our study represents a nearly comprehensive assessment of lipid concentration changes in the gray matter of the human PFC over the entire lifespan, as well as a limited evaluation of lipid concentration changes in three cognitive disorders: SZ, ASD, and DS. Our results reconcile with previous observations, based on substantially smaller numbers of samples, that showed substantial concentration changes in human brain cortex during development [17], as well as aging [8]. Further, our results indicate that lipids identified to correlate with the maximal lifespan duration in mammals [45] experience more temporal concentration changes during the human lifespan (Figure S2). Furthermore, concentration changes along the lifespan detected in our study correlate significantly with expressions of genes interacting with the lipids (Figure S3).

Our analysis, which is based on concentrations of 5024 lipids in a total of 452 human PFC samples, resulted in the following observations:

1. Temporal changes of the cortical brain lipidome form four lifespan stages with boundaries at approximately 100 days, 6 years and 30 years of age. These stages are conserved in chimpanzees and macaques and are traceable at the level of the lipid-interacting gene expression.
2. Cortical aging is not accompanied by an increase in individual variation and numbers of detected compounds, arguing against metabolic damage hypothesis.
3. Cortical aging is characterized by substantial lipidome changes affecting no less than 14% of detected lipids. The majority of these changes (51%) commence during the 50–55-year interval, with differences existing between females and males.
4. Predicted membrane fluidity shows a clear increase over the lifespan.
5. All three cognitive disorders are accompanied by significant lipid concentration changes, which include shared and disease-specific pathway alterations.
6. Genes connected to lipids with decreased concentrations in SZ are significantly overrepresented in genetic variants linked to the disease, indicating the potential role of lipidome changes in SZ etiology.
7. Disease-associated and age-dependent lipidome changes overlap, indicating an older lipidome state in SZ and a younger state in ASD and DS.

### Temporal lipidome stages

Our results indicate that four lipidome lifespan stages are conserved among primates and characterized by differential enrichment in specific lipid classes and pathways, suggesting their relevance to functional changes in PFC organization. The underlying processes might include synaptogenesis and synaptic pruning, as they include a rapid increase of synaptic density in layer II–IV at three months after birth [46], followed by a gradual increase peaking between 5 and 8 years of age [47], followed by synaptic pruning and cortical maturation persisting till 30–40 years of age [47]. The myelination process might also contribute to the temporal lipidome stages, as age-dependent changes of the myelination rate were shown in humans and chimpanzees [48]. At the same time, timing of synaptic maturation and myelination might differ between human and other primate species, even after correction for lifespan differences [46, 48–50]. Thus, further work including determination of lipidome composition markers characteristic of specific cell types and cellular compartments is needed to assess the physiological significance of lipidome lifespan stages.

One noticeable feature characterizing PFC lipidome at different lifespan stages is membrane fluidity. Changes of membrane fluidity are considered to affect functions by influencing cellular material and signaling transmission rates, as well as properties of membrane proteins [2, 51, 52]. Our analysis of the lipidome stages revealed a noticeable stepwise increase in predicted membrane fluidity from the infant to adult stages. While the mechanisms underlying this process are unclear, they might relate to neuron development and differentiation as increased membrane fluidity was reported for human primary neuron cultures [53].

### Lipidome changes in aging

Our study includes 77 samples with ages between 60 and 99 years, 19 of them older than 90, thus providing substantial coverage of the advanced age interval. Yet, we detect no significant increase in numbers of detected lipids and individual variation with increased age, which was predicted by the metabolic damage accumulation hypothesis of aging [37–39, 54]. At the same time, increases in metabolite numbers at advanced age were reported in *Drosophila* [37]. Damage accumulation during aging process at the other molecular levels, including increases in genome instability [55] and transcription variation [56], were reported. Thus, aging-associated heterogeneity increase might be species- and molecular level-dependent.

In contrast to aging, we detected an obvious increase in detected lipid numbers early in life, from newborns to adults, resulting in a 15–20% increase of the PFC lipidome repertoire. Based on the relationship between the lipid white matter enrichment and detection at each stage, we estimate that the contribution of cortical myelination progression to this lipidome repertoire increase is <5%. Yet, in agreement with anatomical data [57], we detect a gradual rise in numbers of lipids preferentially present in white matter with increased age (Table S6).

Overall, 682 lipids showed significant concentration changes in aging. For 51% of them, these changes commence at 50–55 years of age, for 9%—at 65–70 years of age, and for 40%—at 3 months–4 years of age. Notably, the major lipidome transition at 50–55 years matches the transition point of body fat, fat-free mass, and basal energy expenditure trajectories at 52 years [58]. Furthermore, the difference in body composition and energy expenditure trajectories between males and females, with later turning point in males [58], was also observed in our data and grouped specifically in C4 cluster representing aging-related concentration changes of glycerophosphates, glycerophosphoglycerols, glycerophosphoethanolamines, phosphosphingolipids, fatty acyl glycosides, and polyprenols (Fig. 4e and S5). While changes in body composition, metabolic and mitochondrial decline represent well-known hallmarks of aging [59–61], our results indicate that this decline is not uniform and the 50–55-year interval might represent an important global metabolic turn point.

### Lipidome changes in cognitive disorders

Lipid concentration changes were reported for specific lipid classes in many cognitive disorders, including SZ [21–27, 62], ASD [19, 20], DS [18], bipolar disorder [26, 63], and Alzheimer’s disease [64]. In our study, 27 SZ, 17 ASD and five DS patients samples were measured together with the control group in a random order. Overall, despite limited sample numbers, our results confirm the existence of substantial changes in the PFC lipidome composition in all three disorders. Notably, we demonstrate that SZ-associated genetic variants are robustly enriched in genes linked to lipids showing decreased concentrations in SZ in our study. Thus, at least in SZ, lipid concentration changes might be linked to disease causes.

A correction for sample number differences showed a two-fold greater effect of DS on the PFC lipidome compared to SZ and ASD (Figure S6). Still, despite a difference in the overall effect, all three diseases show parallel lipid concentration alterations in two pathways: retrograde eCB signaling and GP metabolism.

Retrograde eCB signaling was shown to play an important role in the control of emotional responses, contextual behavior reactions, and social interactions [65, 66]. Changes in concentrations of eCBs and cannabinoid receptors were reported in SZ [67, 68], ASD [69], and Huntington’s disease [70]. In our analysis, lipids with decreased concentrations in ASD samples in DS1 and DS2 are both enriched in eCB signaling pathway (Figure S7). Of the two well-characterized eCB compounds involved in eCB, arachidonylethanolamide (AEA) shows a concentration decrease in ASD DS2 (Figure S7), consistent with results obtained in model organisms [65, 66]. The second eCB, 2-arachydonoil glycerol (2-AG), shows increased concentration in ASD in both DS1 and DS2, as well as in SZ. Consistently, a 2-AG concentration increase was reported in the PFC of SZ patients [68]. Concentrations of other lipid components of eCB signaling pathway, eCB biosynthesis and degradation products, were not assessed previously. Our results indicate that eCB biosynthesis intermediates, i.e. glycerophosphocholines (PC), glycerophosphoethanolamines (PE), and diacylglycerol (DAG) are mainly decreased in all three disorders, although DAG concentration increases in DS2—the only contradictory result between datasets among seven detected metabolites. The degradation products, i.e. prostaglandin H2 (PG-G) and arachidonate (AA) are decreased and increased respectively (Fig. 5d, Figure S7).

Our results add DS to the list of cognitive disorders characterized by lipid concentration changes in eCB signaling, further supporting the role of this pathway in cognitive dysfunctions. Notably, in aging, we see changes contrasting the DA alterations. Specifically, individuals with no diagnosed cognitive dysfunction show decreased levels of 2-AG and increased concentrations of eCB biosynthesis intermediates starting at ∼51 years of age (Fig. 4d, f). Thus, it is appealing to speculate that these changes might represent a compensatory mechanism counterbalancing the deleterious effects of aging in cognitively healthy individuals.

For the other pathway showing an overall lipid concentration decrease in the three disorders, GP metabolism, lipid concentration decreases and increases were both reported in SZ [21, 23, 25–27, 62]. Our results indicate that, similar to eCB signaling, the disruption of GP metabolism might be a common feature of cognitive dysfunctions. Furthermore, GP metabolism includes metabolism of PCs, PEs, and DAGs participating in eBC biosynthesis. Decreased levels of PCs and PEs were reported in SZ [71, 72]. Thus, the two pathways showing an overall lipid concentration decrease in the disorders are linked.

Among lipidome changes particular to each disease, some could be linked to the reported changes. For instance, a lower concentration of cardiolipin, the critical component of the inner mitochondrial membrane, in SZ (Fig. 5c) matches elevated anti-cardiolipin antibody levels reported in SZ patients [73, 74]. Similarly, elevated levels of triradylglycerols (TG) in DS match the reported overexpression of genes involved in energy consumption and oxidative stress in DS patients [75], as TGs represent one of the major energy sources [76], and were linked to an increase of lipid peroxidation markers [77].

All three of the examined cognitive disorders have an ontogenetic component [78–81]. We observed higher similarities between the lipidome states in ASD/DS patients and cognitively healthy individuals of much younger ages both in terms of content and predicted membrane fluidity properties. Oppositely, the lipidomes of SZ patients were more similar to cognitively healthy individuals of older ages than to age-matched ones. Notably, this observation is consistent with the notion that SZ represents an accelerated aging syndrome, a hypothesis based on higher mortality of SZ patients after correction for psychiatric quality and medical care levels, aging-dependent cognitive decline rates, and increased risk of aging-related diseases, such as diabetes [82]. Accelerated brain aging in SZ patients was also reported based on longitudinal study of MRI-based gray matter density maps [83].

## Conclusions

While lipids represent both the main structural components and important signaling molecules of the human brain, our understanding of their actual involvement in brain functions and dysfunctions is only beginning to emerge. Our study shows that the lipidome composition of the human PFC is highly dynamic, with multiple changes observed in both development and aging, as well as in all three examined cognitive disorders. In the absence of the mature annotation of lipid functionality, analogous to gene-based annotation, interpretation of these changes remains a challenge. Some of the changes, such as the disruption of endocannabinoid signaling in diseases and the presence of opposite changes in aging, the existence of a lipid concentration trajectory breakpoint at ∼55 years of age, the “older” lipidome sate of SZ patients, as well as the connection between a lipid concentration decrease in SZ and genome variants associated with disease, could be connected to previous observations. Other findings, such as the existence of four temporal lipidome stages, differences in predicted membrane fluidity between stages, as well as our observation of no increase in lipidome representation and variability in aging, could not be directly linked to existing knowledge. Altogether, our work sheds light on the lipidome organization of the human PFC and highlights the need for further detailed research of lipidome organization and functions in healthy and diseased brains.

## Electronic supplementary material

supplemental materials

Table S1

Table S2

Table S3

Table S4
