# Supplementary material for: Lipidome alterations in human prefrontal cortex during development, aging, and cognitive disorders
Source: Mol Psychiatry. 2018 Aug 8;25(11):2952–69. doi: 10.1038/s41380-018-0200-8 (PMC7577858; doi:10.1038/s41380-018-0200-8)
Supplement: Supplementary file 1 — supplemental materials [file 41380_2018_200_MOESM1_ESM.docx]

Supplementary Figures


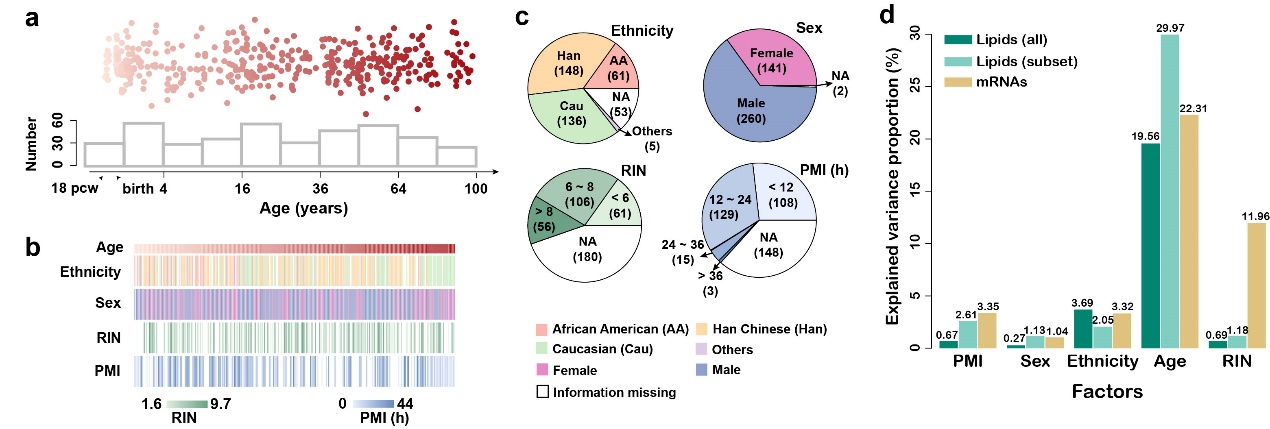


**Figure S1.** Sample information and factor effect. (a) The age distribution of the sampled cognitively healthy human individuals shown as a scatter plot and a histogram. (b) The distribution of sample information – ethnicity, sex, RNA integrity number (RIN), and postmortem interval (PMI) – ordered by individuals’ age. The darker shades correspond to larger values of quantitative variables: age, RIN, and PMI. (c) The pie charts showing sample distribution into groups according to ethnicity, sex, RIN, and PMI information. Numbers in brackets indicate the number of samples within a group. (d) The proportions of the transcriptome and lipidome variation explained by different factors. The colors show datasets: dark green – complete lipidome dataset containing all 403 samples from cognitively healthy individuals; light green – lipidome subset restricted to 72 cognitively healthy individuals used for the transcriptome measurements; light brown –RNA-seq dataset measured in 72 cognitively healthy individuals.

**
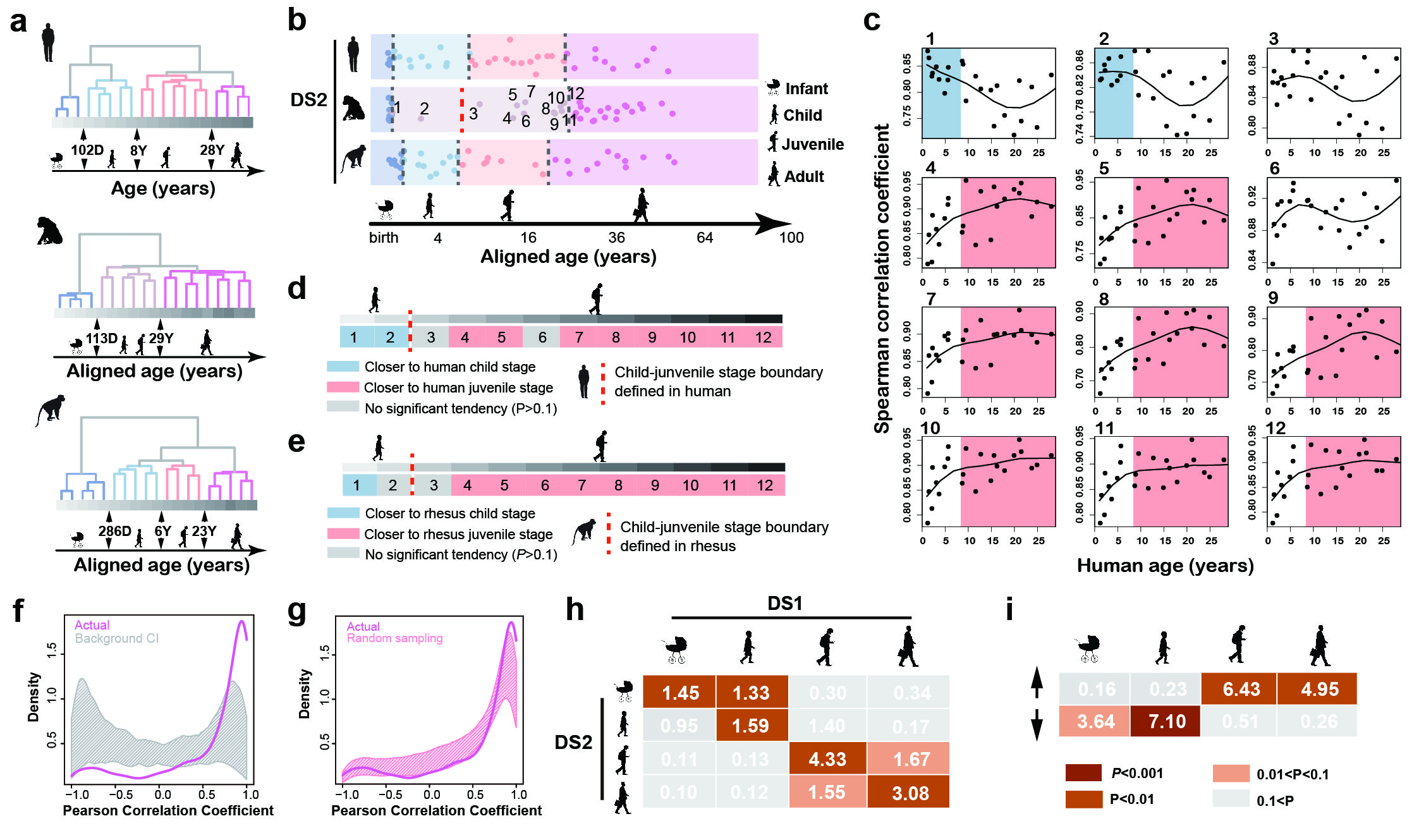
**

**Figure S2**. Consistency of current results with previous lipidome studies. (a) Lifespan stages in DS2 human (top), chimpanzee (middle), and macaque data (bottom). The hieratical clustering was conducted using the (1 – *ρ*) distances calculated based on concentrations of 9,572, 9,370 and 9,572 lipids showing temporal specificity within at least one sliding window in human, chimpanzee and macaque, respectively. The colors mark lifespan stages: blue – infant; light blue – child; orange – juvenile; pink – adult; light brown – child and juvenile combined. The horizontal gray bar indicates the median age in each window, with darker shades corresponding to older age. Numbers show boundary ages (D-days, Y-years). Ages of non-human samples are corrected for differences in species’ maximal longevity. (b) Sample distribution and lifespan stage boundaries calculated using a sliding window approach in human, chimpanzee, and macaque data (DS2). Silhouette symbols indicate species and lifespan stages. Each dot represents a sample. Numbers mark chimpanzee samples located within a single chimpanzee stage representing a combination of child and juvenile stages of humans and macaques. The x-axis shows the square-root-transformed age of assessed individuals. The age of non-human primates was corrected for differences in species’ maximal longevity. The background color represents lifespan stages. (c) Lipid concentration similarities between each chimpanzee sample within the stage representing combined child and juvenile stages and human samples from the child and juvenile stages. Each point shows the Spearman correlation coefficients (*ρ*) calculated using concentrations of 9,370 chimpanzee window-dependent lipids between a chimpanzee sample and a human sample. Numbers above the plots mark chimpanzee samples, as in panel b. Background colors indicate the human stage with a greater similarity to a chimpanzee sample (Wilcoxon rank sum test, nominal *P*<0.1; blue – child stage; red – juvenile stage; none – no significant tendency). The boundary between the child and juvenile stages were the same as in panel b. (d and e) The schematic representation of the similarity estimates between chimpanzee samples from combined stage and human (d) or macaque (e) samples from child and juvenile stages. (f) The distribution of the Pearson correlation coefficients (*r*) calculated between concentration profiles of the 411 DS1-DS2 unambiguously mapped lipids in DS1 and DS2 (pink curve). The gray area covers 95% of the *r* chance distribution calculated by 100 permutations of the samples’ age information. (g) The distributions of the *r* calculated between concentration profiles of the 411 DS1-DS2 unambiguously mapped lipids in DS1 and DS2. The distributions are shown for all DS1 samples (pink curve) and 95% of the 1,000 subsets of 40 DS1 individuals sampled matching the age distribution of DS2 individuals (pink area). (h) Overlap of stage-dependent lipids defined using DS1 and DS2. The numbers represent the odds ratio, calculated as the ratio of the actual number of lipids with differential concentration in the *i*^th^ stage in DS2 (*i*^th^ row) and the *j*^th^ stage in DS1 (*j*^th^ column) versus the median background number calculated by random samplings of the same number of stage-dependent lipids 1,000 times. Silhouette figures indicate lifespan stages with differential lipid concentrations (rows: stages in DS2; columns: stages in DS1). Colors indicate the permutation *P* values calculated in 1,000 permutations of sample labels. (i) Overlap of stage-dependent lipids in DS1 and lipids correlating with species’ maximum lifespan in cortex samples of DS3. Silhouette figures indicate lifespan stages. Arrows indicate directions of concentration change for stage-dependent differences in DS1. Numbers represent the odds ratio and colors indicate the nominal *P* values of hypergeometric tests. The correspondence between colors and *P* values shown by the legend is the same for panel (h) and (i).

**
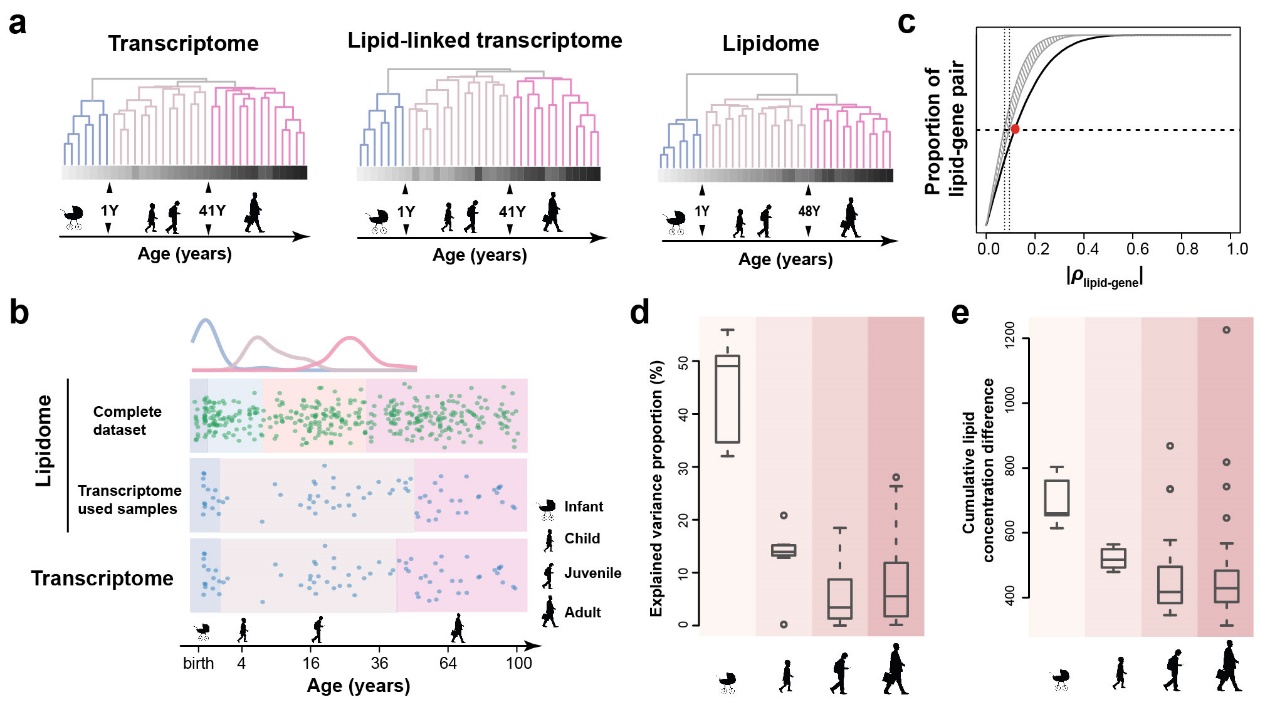
**

**Figure S3**. The correspondence of temporal variation between lipidome and transcriptome datasets. (a) Lifespan stages of human PFC transcriptome and lipidome, based on 72 cognitively healthy human individuals with both transcriptome and lipidome measured. The branch length shows (1 - *ρ*) distances, where *ρ* is a Spearman correlation coefficient of the concentration specificity measures of 15,127 expressed genes (left), 589 expressed lipid-interacting genes (middle), and 4,777 lipids (right) with differential concentrations in at least one sliding window. The branch color shows lifespan stages: blue – infant; light brown – combined child and juvenile stage; pink – adult. The horizontal gray bar indicates the median age in each window with darker shades corresponding to older ages. Silhouette symbols indicate lifespan stages. (b) Lifespan stage boundaries in the complete lipidome dataset, the 72 individual-based transcriptome-matched lipidome dataset, and the transcriptome dataset. Each dot represents a sample. Density curves on top show the variance of boundary age estimates calculated by random subsampling of 200 samples from the complete lipidome dataset 50 times (blue – boundary between infant and child stages, brown – between child and juvenile stages, and pink – between juvenile and adult stages). (c) The proportion of stage-dependent lipid-gene pairs with absolute *ρ* lower than the x-axis cutoff calculated across all cognitively healthy individuals (black curve). The red dot shows the median correlation. The gray area shows the 90% confidence intervals calculated by permutations of samples’ age 100 times. (d) Proportions of the concentration difference variation of the 677 stage-dependent lipids linked to genes in KEGG explained by a differential expression of 936 lipid-interacting genes. The boxplots show the distributions of the squared Pearson correlation coefficients calculated in comparisons between the actual scaled lipid concentrations within a sample and the predicted ones, for all samples within one lifespan stage. (e) Cumulative difference of lipid concentrations within a sample and the average concentration over the lifespan. The boxplots show the cumulative differences calculated by summing up the absolute values of scaled concentrations of the 677 stage-dependent lipids linked to genes in KEGG within a sample, for all samples within one lifespan stage.

**
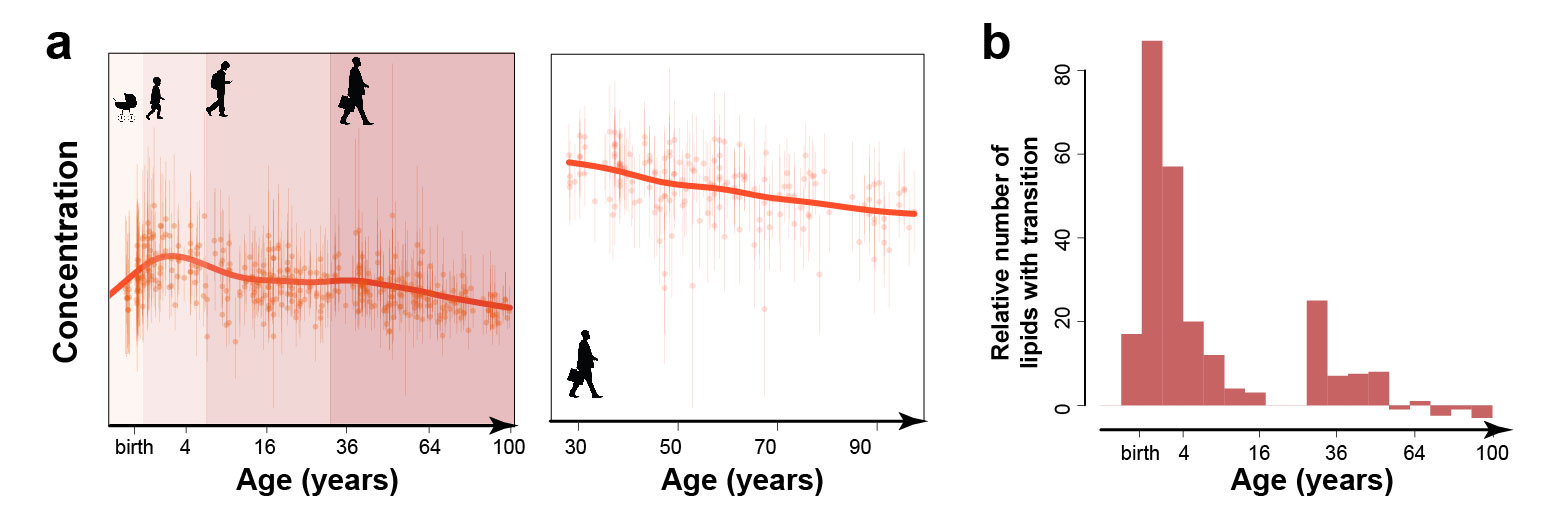
Figure S4**. Temporal concentration pattern of aging-related lipids in cluster C1. (a) Average concentration pattern of 275 aging-related lipids in cluster C1 across the lifespan (left) and during the adult stage (right). Each dot represents the average relative lipid concentration in a sample. The lines represent spline curves built with five degrees of freedom. The stages are indicated by silhouette symbols. (b) Age distribution of transition points of the lipid concentration profiles of aging-related lipids in cluster C1 along the lifespan. The y-axis shows the number of transition points within a given age interval minus the background calculated by permutations of the age labels 100 times.


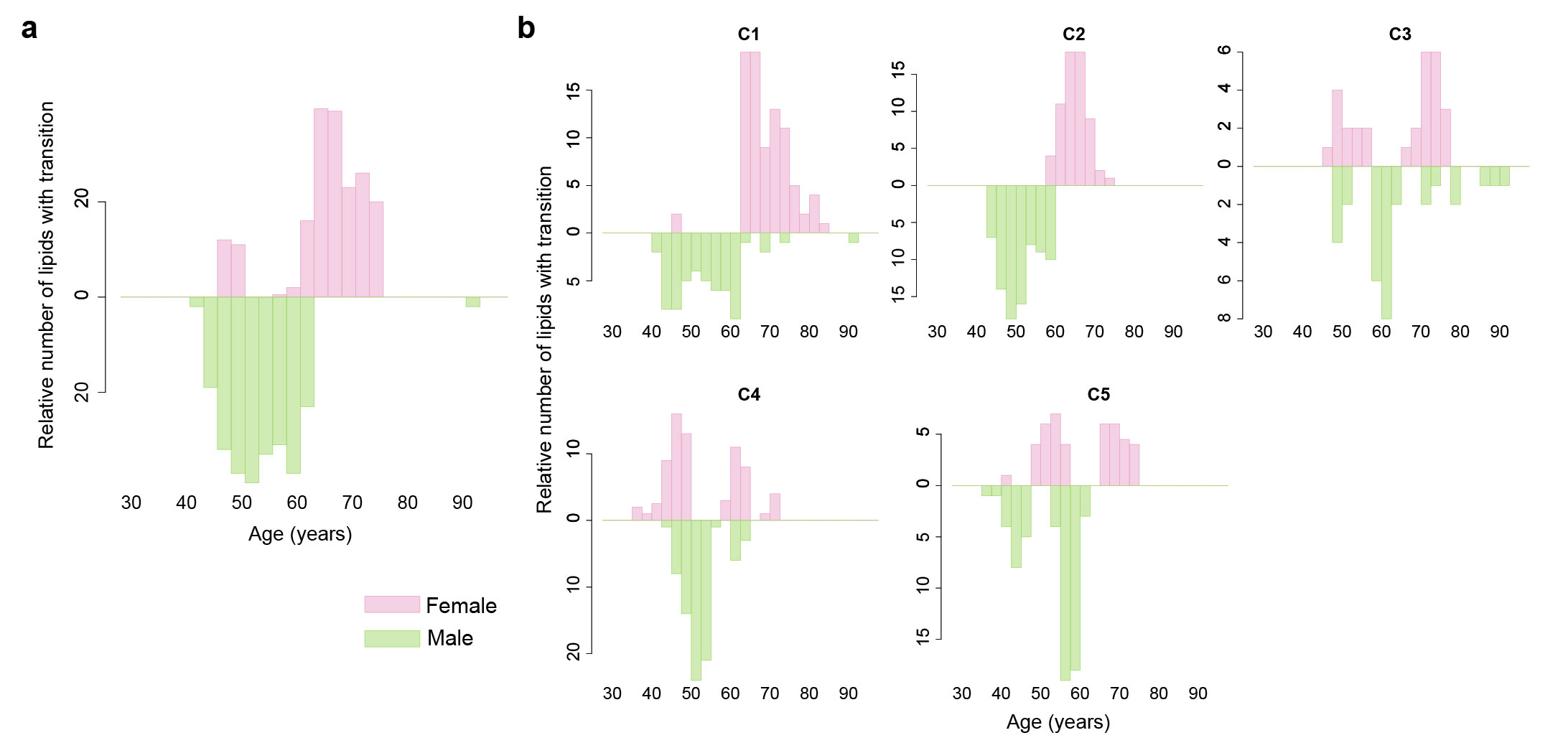


**Figure S5**. Age distribution of transition points of the lipid concentration profiles in the adult stage, based on female and male samples separately. (a) Age distributions of transition points of lipid concentration profiles in the adult stage for aging-related lipids with significant transition points (*N*=524). (b) Age distributions of transition points of lipid concentration profiles for lipids in five aging-related clusters. Colors indicate distributions based on female and male samples: pink – female, green – male. The x-axis shows sample ages. The y-axis shows the number of transitions within a given age interval minus the background calculated by permutations of the age labels 100 times.


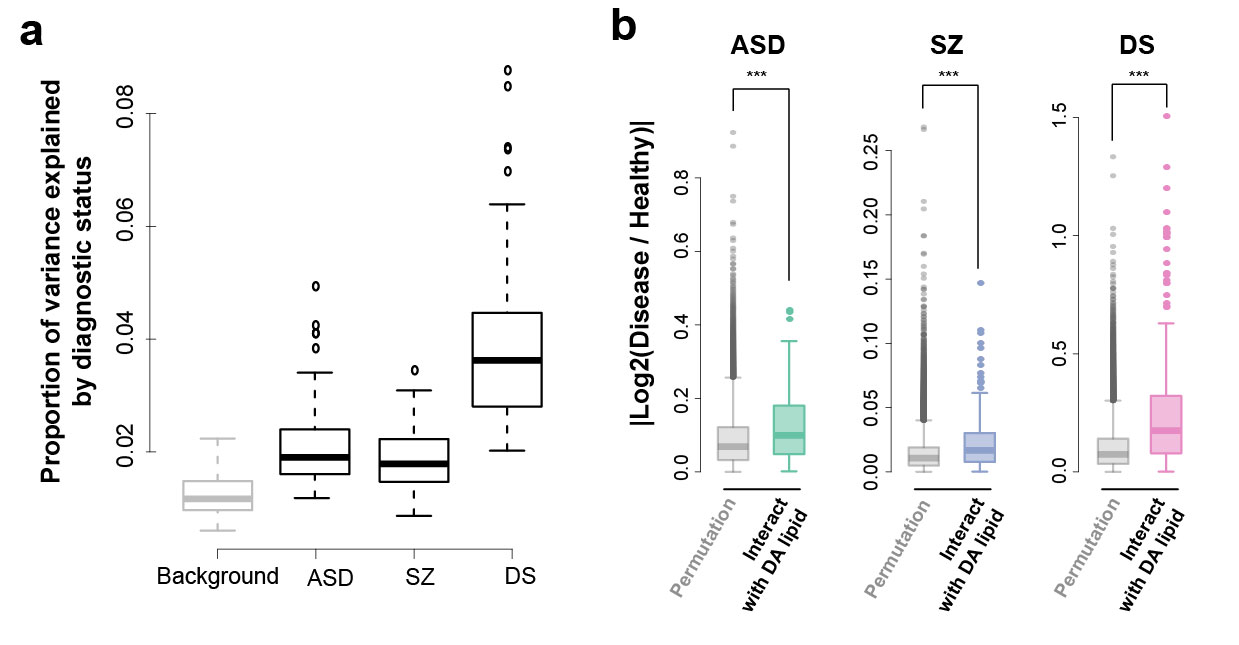


**Figure S6**. Lipidome and transcriptome alterations in the three cognitive diseases. (a) Distributions of the average proportions of lipidome concentration variance explained by diagnostic status, based on bootstrapping over samples with sample size, age, sex, and PMI distributions equalized among three disorders 100 times. The background is estimated based on comparisons of randomly selected samples from the matched control samples to all matched control samples 100 times. (b) Distributions of gene expression differences between disease and control samples, represented by absolute values of log2-transformed fold changes, for genes directly interacting with DA lipids based on KEGG annotation (colored boxes). The gray boxes show background distributions calculated by permuting disease and controls labels 1,000 times. Stars indicate significant differences between distributions in the two-sided Wilcoxon rank sum test (***: nominal *P*<0.001). Gene expression differences were calculated using public datasets retrieved from GEO: ASD-GSE28521, SZ-GSE53978, DS-GSE5390.


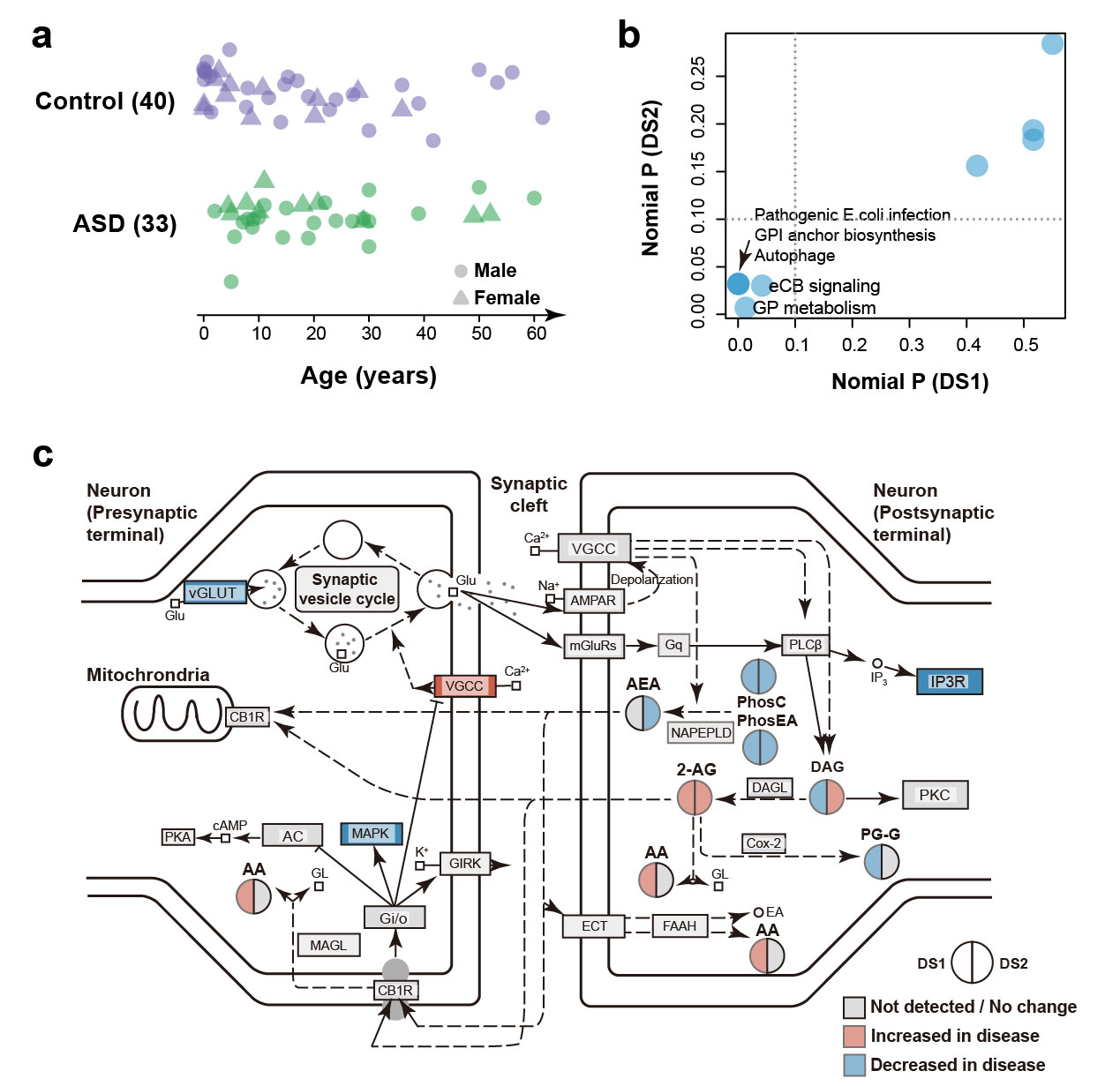


**Figure S7.** Autism associated lipidome changes in DS2. (a) Age distribution of the PFC samples from matched controls (purple) and patients with autism (green) in DS2. Each symbol represents an individual (circle – male, triangle – female). Numbers in brackets show numbers of individuals for each group. (b) Nominal *P* values of hypergeometric test in pathway enrichment analysis calculated based on DS1 (x-axis) and DS2 (y-axis) lipids with decreased concentrations in autism samples identified independently in a respective dataset. Each circle represents one pathway. The black arrow marks three overlapping circles corresponding to three pathways with the same enrichment significance level, with names of these and other pathways enriched in both datasets (nominal *P*<0.1) listed. (c) The schematic representation of autism-associated gene expression and lipid concentration changes in retrograde endocannabinoid (eCB) signaling pathway. The structure of the pathway is based on the KEGG annotation.


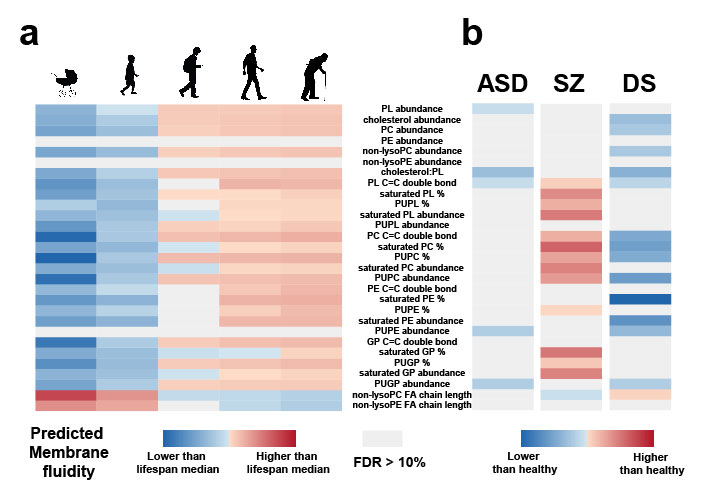


**Figure S8**. Predicted membrane fluidity levels in lifespan stages and disorders. (a) Relative membrane fluidity estimates in lifespan stages. Each row represents a biochemical feature and each column represents one lifespan stage, with the adult stage separated into two intervals: 30-65 years and 65-99 years. The colors indicate the difference between the predicted median membrane fluidity at the given stage and the lifespan median, with the darker shade corresponding to larger differences. Inverted relative concentrations are shown for features which negatively correlate with membrane fluidity: the 2^nd^, 4^th^, 7^th^, 9^th^, 11^th^, 14^th^, 16^th^, 19^th^, 21^st^, 24^th^, 26^th^, 28^th^, and 29^th^ features. Gray indicates the absence of significant concentration differences for a given feature (Wilcoxon rank sum test, BH-corrected *P*>0.1) (b) Relative membrane fluidity estimates in disorders compared to age-matched controls. Colors and features as in panel a.

Supplementary tables

Tables S1-4 are provided as separate excel files.

**Table S1**. Sample information.

**Table S2**. Enrichment of lipid classes and KEGG pathways in stage-dependent lipids.

**Table S3**. Enrichment of lipid classes and KEGG pathways in aging-related lipids.

**Table S4.** Enrichment of lipid classes and KEGG pathways in disorder-associated (DA) lipids.

**Table S5.** Enrichment of genes interacting with lipids showing decreased concentrations in SZ in functional pathways and SZ-associated genetic variants.

| Database | | GRASP database | | | | | | SZDB database |
| --- | --- | --- | --- | --- | --- | --- | --- | --- |
| Genetic association *P* value cutoff^1^ | | *P*<0.05 | | *P*<0.01 | *P*<0.005 | *P*<0.001 | *P*<0.0005 |  |
| *P* value of enrichment^2^ | | 0.0004 | | 0.0193 | 0.0143 | 0.0027 | 0.0573 | 0.0655 |
| KEGG ID | Name of enriched pathway | BH-corrected *P*^3^ | Nominal *P*^3^ | | | | | |
| map  04014 | Ras signaling pathway | 0.0062 | 4.06E-04 | 1.31E-02 | 1.51E-02 | 7.38E-02 | 3.15E-01 | 3.37E-01 |
| map 04015 | Rap1 signaling pathway | 0.0026 | 1.59E-04 | 1.96E-03 | 1.07E-02 | 1.40E-02 | 8.26E-02 | 3.18E-01 |
| map 04062 | Chemokine signaling pathway | 0.0009 | 2.74E-05 | 1.95E-03 | 1.56E-02 | 2.68E-02 | 4.49E-02 | 2.58E-01 |
| map 04070 | Phosphatidylinositol signaling system | 0.0011 | 4.99E-05 | 3.37E-05 | 2.29E-05 | 1.29E-04 | 6.44E-04 | 3.42E-04 |
| map 04072 | Phospholipase D signaling pathway | 0.0096 | 9.17E-04 | 5.08E-03 | 8.59E-04 | 1.45E-02 | 5.17E-02 | 1.06E-02 |
| map 04650 | Natural killer cell mediated cytotoxicity | 0.0068 | 4.79E-04 | 1.09E-03 | 1.74E-02 | 4.32E-02 | 4.43E-01 | 1.00E+00 |
| map 04660 | T cell receptor signaling pathway | 0.0011 | 4.82E-05 | 8.37E-03 | 2.89E-02 | 9.94E-02 | 3.22E-01 | 1.00E+00 |
| map 04664 | Fc epsilon RI signaling pathway | 0.0009 | 2.43E-05 | 7.04E-04 | 1.83E-03 | 3.59E-02 | 4.21E-01 | 1.00E+00 |
| map 04666 | Fc gamma R-mediated phagocytosis | 0.0009 | 1.69E-05 | 7.08E-03 | 3.66E-02 | 5.30E-02 | 2.72E-01 | 3.08E-01 |
| map 04724 | Glutamatergic synapse | 0.0010 | 3.57E-05 | 2.44E-03 | 7.41E-03 | 2.34E-02 | 7.14E-02 | 1.49E-01 |
| map 04725 | Cholinergic synapse | 0.0079 | 5.96E-04 | 8.94E-04 | 3.49E-03 | 1.22E-03 | 5.10E-02 | 1.26E-01 |
| map 04730 | Long-term depression | 0.0096 | 8.18E-04 | 4.32E-04 | 1.24E-03 | 2.93E-02 | 8.25E-02 | 1.61E-01 |
| map 04750 | Inflammatory mediator regulation of TRP channels | 0.0011 | 5.28E-05 | 4.42E-04 | 5.93E-04 | 8.40E-04 | 8.30E-03 | 3.33E-02 |
| map 04912 | GnRH signaling pathway | 0.0009 | 2.43E-05 | 7.04E-04 | 1.83E-03 | 3.59E-02 | 9.41E-02 | 1.72E-01 |
| map 04916 | Melanogenesis | 0.0096 | 9.05E-04 | 2.29E-04 | 1.29E-03 | 6.91E-03 | 3.32E-02 | 1.02E-01 |
| map 04919 | Thyroid hormone signaling pathway | 0.0015 | 8.09E-05 | 5.63E-05 | 1.01E-04 | 1.81E-03 | 2.63E-02 | 2.16E-01 |
| map 04925 | Aldosterone synthesis and secretion | 0.0096 | 9.20E-04 | 1.63E-03 | 3.58E-03 | 9.97E-04 | 1.89E-02 | 1.94E-01 |
| map 04933 | AGE-RAGE signaling pathway in diabetic complications | 0.0009 | 9.90E-06 | 5.63E-05 | 1.01E-04 | 1.88E-04 | 3.25E-03 | 2.16E-01 |
| map 05231 | Choline metabolism in cancer | 0.0003 | 1.47E-06 | 4.91E-04 | 2.73E-03 | 6.64E-02 | 3.01E-01 | 5.14E-02 |
| Consistency in pathway enrichment based on different SZ-associated gene lists^4^ | | | | 1.71E-09 | 3.16E-09 | 6.53E-08 | 1.57E-08 | 8.42E-06 |

^1^ Cutoffs of *P* value provided by GRASP database in genome-wide association studies to define SZ-associated SNP. The nearest gene of the SNP provided by the database is defined as the SZ-associated gene.

^2^ *P* values of hypergeometric tests used to determine the enrichment of SZ-associated gene in genes interacting with lipids showing decreased concentrations in SZ.

^3^ *P* values of hypergeometric tests used to determine the enrichment of KEGG pathways in SZ-associated genes interacting with lipids showing decreased concentrations in SZ. Enriched pathways are defined as those with BH-corrected-*P*<0.01, with SZ-associated genes defined as those with *P*<0.05 in GRASP database.

^4^ *P* values of one-sided Wilcoxon rank sum tests used to test whether the enriched pathway defined above shows stronger enrichment than all pathways linked to genes interacting with robustly detected lipids, based on different lists of SZ-associated genes.

**Table S6.** White matter enrichment of stage-specific detected lipids.

|  | | Undetected stage | | |
| --- | --- | --- | --- | --- |
|  |  | Infant | Child | Juvenile |
| Detected stage^2^ | Child | 0.84^1^ (-0.25/-0.13) |  |  |
|  | Juvenile | 0.69 (-0.21/-0.11) | 0.122 (0.09/-0.18) |  |
|  | Adult | 0.6 (-0.17/-0.10) | 0.053 (0.11/-0.17) | 0.104 (0.20/-0.13) |

^1^ Nominal *P* values of one-sided Wilcoxon rank sum tests used to compare white matter enrichment of stage-specific detected lipids to that of non-specific ones. Values in brackets show the median white matter enrichment of stage-specific detected lipids (left) and that of non-specific ones (right). Larger values represent stronger white matter enrichment. White matter enrichment was defined as the log10-transformed fold change of concentration in white matter to concentration in gray matter. The color of cells indicate the direction of enrichment. Orange indicates that stage-specific detected lipids has stronger white matter enrichment than non-specific ones, while blue indicates weaker enrichment. A darker shade indicates a greater degree of enrichment.

^2^ Stage-specific detected lipids were defined as the lipids that were not detected in one stage (shown by columns), but were detected at a later stage (shown by rows), while non-specific lipids were defined as those detected at both stages. A lipid was classified as detected at one stage if it was detected in over half of the samples in this stage.
